# Dr. Kinglake, on the Treatment of Burns and Scalds

**Published:** 1807-09

**Authors:** Robert Kinglake

**Affiliations:** Taunton


### *To the Editors of the Medical and Physical Journal.*

Gentlemen,

YOUR facetious Correspondent, designated by the letters T. Y. has, in a late number of your Journal, indulged, at no small length, in sportive comments on the heating and cooling methods of treating Burns and Scalds. Well timed *wit* must ever have its due effect; unenviable indeed would be the insensibility that could be *dead* to its pleasing influence, when seasonably employed. It would be difficult, however, to specify on what occasion *wit* could be either *well-timed* or admissible on a subject of medical investigation. Your Correspondent, therefore, has not been strictly judicious in adopting the ludicrous style of discussion displayed in the Paper referred to; nor has he been more correct in instituting a scheme of experiment that necessarily must be nugatory, from the incongruous circumstances in which it was conducted.

It is not compatible with any known law of motive power obtaining in the animal economy, for any part of the system so far to admit of the agency of contrary powers, as to produce a distinctness of effect, accurately corresponding to the action of the different causes. The idea is indeed a paradox, and the operation of such opposing influence must go either wholly to annul, or greatly to diminish the respective efficiencies of either agent. Heat and cold are not the same states; they are in fact, different conditions of the same thing, and necessarily destructive of each other.

Your

Your Correspondent might surely have an opportunity of gratifying his anxiety for solving the question respecting the comparative advantages of the heating and cooling treatment of burns and scalds, by making his experiments on different subjects. Occasions for trying the effects of remedies in such cases are not rare; they present in ample abundance, and will, no doubt, hereafter, enable your correspondent satisfactorily to ascertain whether injuries from fire are least advantageously cured by heating or cooling applications. This comparative trial has been already made in some degree, and has been extended to another instance, in the number of cases here subjoined, of the benefits resulting from topical refrigeration in burns and scalds.

Dr. Evans, whose situation at Ketley affords him superior practical opportunities for putting the subject in question to the test of experiment, has furnished me with the following additional examples of the salutary efficacy of the cooling treatment. Many similar proofs of its curative power have been before transmitted to me by the same respectable author, which I have already communicated to the public through the medium of your Journal. The further accession of intelligence contained in the subsequent cases, will of course enhance the value of the preceding, and collectively afford a mass of evidence on the subject, sufficiently important to be highly deserving of public attention.

It is worthy of remark, that Dr. Evans, in his several communications to me on the subject in discussion, has not mentioned a case in which the cooling treatment did not succeed to his wishes. If any had occurred, his regard for truth, his ingenousness, and the claims of impartial inquiry, must have irresistably disposed him to have made it known. What therefore has hitherto obtained so uniformly, may be justly expected to continue, as a natural order of things, not to be controverted by mere declamation, whether *lightly* or gravely conducted.

I am very solicitous that the pretensions of the *terebinthinate* remedy should be examined with the utmost practical correctness, and have been desirous of adequate occasions for putting it to the proof of actual experiment; but as yet, my own experience of its powers does not enable me to deliver a decisive opinion on its real merits. Time, the infallible arbiter of doubt, will eventually settle the question at issue between the opposite modes of treatment. It is erroneous to endeavour to accelerate the probation in a manner inconsistent with the slow attainment of truth. The real state of things in ambiguous circumstances cumstances cannot be speedily discovered; it is only by cautious, patient, and unremitted research, that such information can be ultimately acquired.

In the fullest confidence that persevering investigation will sooner or later indisputably confirm the superior practical value of the cooling treatment of burns and scalds, its advocates need not be discouraged by any unphilosophical objections that may be made against it, whether proceeding from the *comic* levity of your correspondent T. Y. or from the more *decorous* opposition of those who conceive it ought to be superseded by *terebinthinate* efficacy. I am, &c.

*Taunton, August* 6, 1807. ROBERT KINGLAKE.

---

*Additional Cases of the beneficial Effects of the Cooling Treatment of Burns and Scalds; communicated in a Letter to* Dr. Kinglake, *by* Dr. Evans.

"Dear Sir,

"In compliance with your request some months since, I now transmit you a farther account of the good effects of the cooling treatment of Burns and Scalds; which, with the numerous instances of similar efficacy already before the public, will, I trust, be sufficient to induce every unbiassed practitioner to give it a fair trial. The cold application should be continued as long as any sensation of pain remains. The result, in many cases, will be the formation of a new surface, with little or no ulceration on the removal of the old one. I generally puncture the blisters with the point of a needle, which diminishes the pain by lessening the tension of the skin.

"If this communication affords any information that you think worthy of notice, you are at liberty to make such use of it as you may judge proper.

"I am, dear Sir,

"Your's respectfully,

*Ketley, July* 29, 1807. "J. Evans.

"June 16th, 1804.—William Dudlick and John Jones, two boys, about 14 years of age each, had their faces and hands severely burnt by the explosion of gunpowder, and were both cured in a week by the cooling treatment, without any other application.

"Oct. 10th, 1806.—John Turner, aged 24, had the whole of his face burnt by the explosion of gunpowder; considerable tumefaction with numerous vesications took place; the cooling plan of treatment procured immediate ease, and the patient was cured in a week without ulceration.

"Oct

"Oct. 26th, 1806.—Samuel James, aged 40, had his face, hands, and back most severely burnt by the explosion of hydrogen gas in a coal mine. The cold application was used to the face and hands, and warm oil of turpentine, according to Dr. Kentish's plan, (originally recommended by Heister) was applied to the back, and dressed afterwards with the unguent. resinæ flav. softened down with the same, in order to try which mode of treatment afforded the most immediate ease to the patient, as well as the mosst expeditious cure. According to the patient's own account, the pain of the hands and face was immediately relieved by the cold application, but he complained of the oil of turpentine occasioning a smarting sensation on the back for five or six hours. This mode of dressing was continued for the space of two days; but observing a considerable degree of inflammation remaining from the terebinthinate application, that dressing was changed for the neutralized cerate, which the patient did not observe (his eyes being closed by the great tumefaction of the face), but he expressed the utmost satisfaction from the superior comfort he felt in that dressing, compared with the former. The next day the back appeared much less inflamed, continued gradually getting better, and was cured in three weeks. I am confident the back would have gotten well sooner under the cooling plan of treatment, for the patient constantly complained of great heat in the part during the application of the oil of turpentine.*

"William Cox, aged 55, had his face, ears, and hands severely burnt at the same time. Immediate ease was afforded by the cooling treatment. The face was cured in a week, notwithstanding there were several large vesications; and the hands, after a slight ulceration, were cured in a fortnight by the neutralized cerate.

"Dec. 11th, 1806.—Joseph Trenter, had his face and arms, with part of his back, burnt in a coal mine, by the explosion of hydrogen gas. He experienced the same good

* Although (as before observed) making a comparative trial of the heating and cooling modes of treatment in the same person is not physiologically correct, and that, therefore, the result of such procedure must be too much vitiated to be conclusive; yet, the case here recited, clearly demonstrates the beneficial influence of the cold application in alleviating pain, and the noxious effects of the heating quality of turpentine in augmenting it. Heating and cooling agents have necessarily a contrary operation, so that their respective action must be rather confounded than distinctly explained by being conjointly employed in the same patient. The interchange of effect reciprocated by such constrasted agency is undoubtedly good effects from the use of cold application, as the above mentioned patients, and was cured in sixteen days.

"Nov. 30th, 1806.—Elizabeth Richards, a young woman, servant in a gentleman's family in this place, had her foot and part of her leg scalded, by accidentally stepping into a bucket half full of boiling water, which had been carelesly left at the bottom of some steps leading from one room to another, and being in the dusk of the evening, she was not aware of her danger. The injured foot and leg were immediately plunged into a bucket full of cold water, and retained therein until bed time, which afforded perfect ease. Linen rags were constantly applied wet with cold water during the night, and renewed, when required by any sensation of pain, during nine days. The patient was cured in a fortnight, with little ulceration; none indeed would have taken place, had it not been for the hastiness of a fellow servant in stripping off the stocking immediately after the accident, to which some of the skin adhered.

"June 23d, 1807.—Ann Parish, a corpulent woman, aged 60, in carrying a pail full of boiling water upon her head, struck her foot against a rail that lay on the ground, in consequence of which she fell and scalded her face, arm and breast. The application of cold water gave her immediate ease. It was continued as long as any inflammation or pain remained. The face was cured without the least ulceration. The arm in the fall, coming forcibly in contact with the ground, suffered a loss of skin from the wrist to the elbow, notwithstanding it was cured in a fortnight. The breast was sometime longer in cure, being more deeply ulcerated. J. Evans."

---

### *To the Editors of the Medical and Physical Journal.*

Gentlemen,

In the second Volume of Fox's Work on the Teeth, recently published, mention is made of *a new method of stopping teeth* by means of "the fusible," (or Newton's) "metal," which had been suggested to Mr. F. by "some chemical

---

doubtedly very considerable, but much too indefinitive to admit ot being estimated with any degree of practical precision. The testimony, however, here adduced, brings into question the justness of T. Y's report, that fewer inconveniences arose from the use of *turpentine* than from that of *cold water*. Dr. K.

